# Decoding the Hydrogen-Transfer Synergy in *Camellia oleifera* Shell Hydrochar/HDPE Co-Pyrolysis: A Kinetic and Thermodynamic Perspective

**DOI:** 10.3390/polym18192423

**Published:** 2026-10-04

**Authors:** Akash Kumar, Lata Kumari, Asma Leghari, Azhar Ali Laghari, Imtiaz Ali Jamro, Moses Akintayo Aborisade, Belay Tafa Oba, Qiuxia Meng, Qiang Zhang

**Affiliations:** 1 College of Resource & Environment, Shanxi Agricultural University, Taiyuan 030031, China; 2 Institute of Eco-Environment and Industrial Technology, Shanxi Agricultural University, Taiyuan 030031, China; 3 Soil Health Laboratory in Shanxi Province, Taiyuan 030031, China; 4 School of Chemical Engineering of Technology, Tianjin University, Tianjin 300350, China; 5 School of Energy and Power Engineering, Shandong University, Jinan 250061, China; 6 Osmani & Company (Pvt.) Ltd., Karachi 75400, Pakistan; 7 School of Civil Engineering, Guangzhou University, Guangzhou 510006, China; 8 College of Natural Science, Arba Minch University, Arba Minch 21, Ethiopia; 9 Shanxi Agricultural University, Taiyuan 030031, China

**Keywords:** biomass valorization, co-pyrolysis, hydrochar, plastic waste conversion, isoconversional kinetics, sustainable waste-to-energy process

## Abstract

This study examined the co-pyrolysis of high-density polyethylene (HDPE) and hydrochar from *Camellia oleifera* shell (HCS), focusing on thermal decomposition behavior, non-isothermal kinetics, thermodynamics, and the interaction mechanism between the two feedstocks. Hydrothermal carbonization raised the heating value of *Camellia oleifera* shell from 18.07 MJ/kg (raw CS) to 20.80 MJ/kg (HCS), confirming its fuel-upgrading effect, while HDPE alone reached 46.40 MJ/kg. TG-DTG analysis of HDPE, HCS, and their blends at five HCS:HDPE mass ratios (1:9, 3:7, 5:5, 7:3, and 9:1, denoted 1HCS9HDPE through 9HCS1HDPE) at 5–25 °C/min showed a single sharp decomposition stage for HDPE, a broader profile with much higher char yield for HCS, and non-additive DTG behavior in the blends, pointing to a synergistic interaction. Isoconversional kinetics (FWO, KAS, Starink, Friedman) showed activation energy rising steadily with conversion for HDPE (170–240 kJ/mol), a plateau-then-sharp-rise pattern for HCS (180–400 kJ/mol), and marked fluctuations at intermediate conversion for the balanced blends, a signature of composition-dependent synergy. Thermodynamic analysis (ΔH, ΔG, ΔS) confirmed an endothermic, non-spontaneous process throughout, with entropy trends mirroring the kinetic fluctuations. A hydrogen-radical transfer mechanism between HDPE- and hydrochar-derived intermediates is proposed to explain the reduced char yield and non-additive behavior of the blends. Overall, the results support hydrochar–HDPE co-pyrolysis as a viable route for combined agricultural and plastic waste valorization and provide kinetic and mechanistic data to guide co-pyrolysis process design.

## 1. Introduction

Recent global challenges of plastic pollution and accumulation of agricultural and agribusiness waste have increased the search for integrated thermochemical valorization strategies that can address both streams together at the same time. Plastic production has grown rapidly, but only a small percentage of plastic waste produced is actually recycled; the rest is landfilled, burned or poorly managed and released into the environment [1,2]. HDPE is one of the most widely produced and discarded olefin commodity plastics and because it is both non-biodegradable and inert at ambient conditions it persists in the environment for decades once released and recovery of this material through routes that use energy and resources efficiently is a high priority [3]. At the same time, residues from agriculture and forestry are produced in similarly huge quantities worldwide, and burning openly or uncontrolled decomposition is a major source of greenhouse gas emissions and contamination of soil and water. *Camellia oleifera* shell (CS) is a lignocellulosic byproduct resulting from oil extraction from *Camellia oleifera*, one of the four major woody oil crops in the world. This shell makes up a huge percentage of fresh fruit mass and China alone generates several million tons of CS as waste [4,5]. Currently, most of this residue is discarded quickly or burned for disposal; this represents both lost bioenergy and avoidable environmental burden [6]. Pyrolysis has emerged as one of the most promising thermochemical conversion technologies for simultaneously dealing with plastic and biomass waste streams because it is relatively simple, flexible in handling feedstock and capable of producing valuable solid (char), liquid (bio-oil/wax) and gas products without requiring investment similar to other routes for converting waste into energy [7]. Co-pyrolysis, which means processing both biomass and plastic waste together in a single reactor at the same time, has attracted particular research interest because interaction between the two feedstocks during decomposition produces synergistic effects that cannot be achieved if each material is processed separately. Specifically, hydrogen-rich nature of polymers such as high-density polyethylene allows them to act as in situ donors of hydrogen to radicals rich in oxygen that result from decomposition of biomass. This improves deoxygenation, suppresses repolymerization and formation of char and enhances yield and quality of liquid products from pyrolysis. At the same time, components of biomass also promote fluidization of plastic and reduce formation of coke or blockages during plastic pyrolysis [8,9].

Recent work has also looked at pyrolyzing different raw lignocellulosic materials together with polyolefins such as high-density polyethylene, low-density polyethylene, and polypropylene. Results using techniques like thermogravimetry, kinetics and distribution of products generally show positive synergistic interactions [10,11]. However, most studies that have looked at this have used untreated raw biomass as co feedstock. Attention to pyrolyzing plastics together with hydrochar, which is a solid product from hydrothermal carbonization (HTC), has been very limited despite HTC becoming a more attractive pretreatment for wet or hard-to-dry biomass because HTC operates at moderate temperature and autogenous pressure and produces material with higher carbon content, lower ash and volatiles and better thermal stability and energy density compared to parent biomass [12,13]. Because HTC removes much of the readily hydrolyzable hemicellulose fraction and partially aromatizes the residual lignin structure, the resulting hydrochar has a very different thermal decomposition profile and this difference also means different synergistic behavior with plastics that have been pyrolyzed together but this distinction has not been studied much in the kinetics literature [13,14]. Despite the individual literature that studies synergy between biomass and plastic (i) pyrolysis together, (ii) HT production and characterization, and (iii) valorization of shells of *Camellia oleifera* directly, there has not yet been a comprehensive and systematic study that looks at behavior of co-pyrolysis, kinetics at multiple heating rates, thermodynamics and reaction mechanism blending hydrochars made from *Camellia oleifera* shells with HDPE. Specifically, there is still a lack of understanding of how chemical structure changes in hydrochar compared to raw biomass affect interaction synergistically across a full range of blending ratios. We do not understand how this interaction manifests in activation energy via multiple isoconversional methods and thermodynamic parameters such as ΔH, ΔG and ΔS. We also do not understand what mechanism controls the observed behavior. Addressing this gap is important both for advancing the fundamental chemistry of hydrochar plastic co-pyrolysis and for designing reactors that integrate agricultural waste and plastic waste processing practically.

Considering this gap, objectives of this study are to: (1) Study thermal decomposition behavior of HDPE and *Camellia oleifera* shell hydrochar (HCS) and blends at five different mass ratios (1 HCS:9 HDPE, 3 HCS:7 HDPE, 5 HCS:5 HDPE, 7 HCS:3 HDPE, and 9 HCS:1 HDPE) using TG (Thermogravimetry) and DTG (Derivative Thermogravimetry) analysis at different heating rates ranging from 5 to 25 °C/min (2) Evaluate kinetics of individual feedstocks and blends non isothermally using multiple free model methods (FWO, KAS, Starink and Friedman) to determine conversion dependent activation energy (E_a_) and evidence of synergistic (non-additive) kinetic behavior is also sought. (3) Determine activation thermodynamic parameters (ΔH, ΔG, ΔS) for all samples using Eyring theory of activated complexes and relate trends of conversion to underlying decomposition chemistry. (4) Propose and discuss a plausible reaction mechanism for co-pyrolysis of HCS and HDPE integrating findings from TG DTG, kinetic and thermodynamic work to explain the observed synergistic interaction between hydrochar and components of HDPE.

## 2. Materials and Methods

HDPE was sourced from Jinan, Shandong and supplied as is. *Camellia oleifera* shells (CS) were sourced from Tianjin, China, and washed in deionized water to remove surface contaminants. They were then oven-dried at 105 °C for 24 h before use.

### 2.1. Preparation of Hydrochar (HCS)

The preparation of hydrochar was undertaken via hydrothermal carbonization (HTC) of dried *Camellia oleifera* shell (HCS). In this work, HCS was produced at Tianjin University (Tianjin, China) using a 600 mL hydrothermal reactor (HT–250 J0, HTLAB, Beijing, China). For each batch, 20 g of raw *Camellia oleifera* shell and 200 mL of deionized water (solid-to-liquid ratio 1:10) were charged into the reactor vessel and thoroughly mixed. After sealing, the reactor was purged with argon to eliminate oxygen from the system. The mixture was stirred at 150 rpm and heated to 180 °C, then held at this temperature for 30 min; pressure inside the vessel was generated autogenously by the water vapor produced under these conditions. Once the reaction period elapsed, the reactor was left to cool naturally to room temperature before the solid product was recovered by vacuum filtration. The isolated hydrochar was then dried in an oven at 105 °C for 24 h prior to use in subsequent pyrolysis testing.

The hydrochar yield was calculated as:

(1) $$\mathrm{Yield}\;\left(\mathrm{wt}.\%\right)=\frac{\mathrm{mass}\;\mathrm{of}\;\mathrm{hydrochar}}{\mathrm{mass}\;\mathrm{of}\;\mathrm{dry}\;\mathrm{raw}\;\mathrm{feedstock}}\times 100\%$$

The hydrochar yield for HCS, prepared at 180 °C with a 30 min residence time (solid-to-liquid ratio of 1:10), was 32.35 ± 0.85 wt.%. Following drying, HCS was ground and sieved to a particle size of <0.15 mm, matching the particle size used for HDPE and raw CS (Section 2.3) to ensure comparable heat and mass transfer conditions across all samples during TGA.

### 2.2. Sample Blending

Prior to blending, HDPE pellets were cryo-milled/ground and sieved to a particle size of <0.15 mm, and HCS was similarly ground and sieved to the same particle size range <0.15 mm to minimize particle-size-related heterogeneity between the two components. HDPE and HCS were combined at the target mass ratios (1:9, 3:7, 5:5, 7:3, and 9:1, HCS: HDPE) and dry-mixed using a vortex mixer for 10 min to ensure homogeneous distribution of the two components. Given the small sample mass of 10 mg used for each TGA run, each blend was mixed in a larger batch of 2–5 g prior to sub-sampling, and five random sub-samples were weighed and visually checked for consistency by comparing repeat TGA mass-loss curves to confirm mixing uniformity before proceeding to the pyrolysis experiments.

### 2.3. Feedstock Characterization

The proximate composition (ash content, moisture content, volatile matter content, fixed carbon content) of samples was determined using ASTM D3172–D3175/GB/T 28731 standards [15,16,17,18,19]. Elemental (ultimate) analysis (C, H, N, S; O by difference) of samples was completed using a CHNS analyser model. The higher heating values (HHVs) of samples were calculated based on elemental analysis data as shown in Table 1.

### 2.4. Thermogravimetric Analysis (TG-DTG)

Pyrolysis tests were undertaken on the samples under non-isothermal conditions using a TGA instrument. Each test involved loading approximately 10 mg of sample (individual component, blend, etc.) into the TGA. Samples were then subjected to five different heating regimes at rates of 5, 10, 15, 20 and 25 °C/min. Each test was conducted in an inert atmosphere at a flow rate of 50 mL/min using nitrogen gas (N_2_). A continuous record of TG (mass loss vs. temperature) and DTG (rate of mass loss vs. temperature) was generated during each experiment. Three replicate runs were undertaken on all samples to verify the repeatability of results.

To quantify the synergistic effect, the theoretical (mass-weighted) TG and DTG curves of each blend were calculated from the individually measured curves of HDPE and HCS under identical conditions.

*W_cal_*(*T*) = *x_HDPE_* × *W_HDPE_*(*T*) + *x_HCS_* × *W_HCS_*(*T*) (2)

where W_calc_(T) is the calculated (theoretical) mass fraction remaining at temperature T, and X_HDPE_ and X_HCS_ are the mass fractions of HDPE and HCS in the blend, respectively. The deviation between the experimental and calculated curves, ΔW, was used to quantify the degree and direction of the synergistic effect.

Δ*W*(*T*) = *W*_exp_(*T*) − *W_calc_*(*T*) (3)

### 2.5. Kinetic Analysis (Isoconversional Methods)

To determine the perceived activation energy (E_a_) of pyrolytic decomposition, the FWO, KAS and STK models were applied. To determine the apparent activation energy (E_a_) of pyrolytic decomposition, the FWO [20], KAS [21], and Starink [22] models were applied, following the model-free isoconversional procedures recommended by the ICTAC Kinetics Committee for the reliable determination of activation energy from thermal analysis data [23]. The linearity and goodness-of-fit of the isoconversional plots for all methods and conversion steps (α = 0.1–0.9) were evaluated via the coefficient of determination (R^2^). The governing equations are:

The pyrolytic kinetics were determined based on the thermogravimetric data, thus:

The conversion degree (α) can be specified as follows:

(4) $$\alpha =\frac{m_{o}-m_{t}}{m_{o}-m_{f}}$$

where *m_t_* is the sample at time t, and *m_o_* and *m_f_* are the initial and final weights, respectively.

The conversion rate is then expressed as

(5) $$\frac{d\alpha }{\mathrm{dt}}=k(T)\cdot f(\alpha )$$

where the symbol *k*(*T*) denotes the reaction rate constant, which follows the Arrhenius equation, accounting for the temperature dependence, and f(α) represents the reaction mechanism model.

(6) $$k(T)=Ae^{-\frac{E_{a}}{\mathrm{RT}}}$$

(7) $$\beta \frac{d\alpha }{\mathrm{dT}}=Ae^{-\frac{E_{a}}{\mathrm{RT}}}f\left(\alpha \right)$$

The integrated form of Equation (5) is given by:

(8) $$G(\alpha )=\int_{0}^{\alpha }\frac{d\alpha }{f(\alpha )}=\int_{T0}^{T}\left(\frac{A}{\beta }\right)\exp\left(\frac{-E_{a}}{\mathrm{RT}}\right)\mathrm{dT}=\left(\frac{AE_{a}}{\beta R}\right)p(u)$$

where T_0_ is the initial temperature of the pyrolysis run. Following standard practice in non-isothermal kinetics, the lower limit of the temperature integral is commonly approximated as zero, since the integrand Ae^−E_a_^^/RT^ is negligibly small at temperatures well below the onset of decomposition; this approximation introduces negligible numerical error and does not imply integration from absolute zero, which is physically unattainable.

Here, u = E_a_/RT, and p(u) is the temperature integral approximation function, for which no closed-form analytical solution exists; various approximations of p(u) give rise to the different integral isoconversional methods (FWO, KAS, Starink) used in this study.

#### Iso-Conversional Methods

The activation energy (*E_a_*) was estimated using three model-free iso-conversional methods: FWO [20], KAS [24], and STK [22]. By substituting *u* = *E*/*RT* into the fundamental kinetic equation, different approximations for the temperature integral *p*(*u*) allow for the determination of *E_a_* without assuming a reaction model *f*(*α*). Using Doyle’s approximation [25],

(9) $$\log p(u)=-2.315-0.4567\frac{E_{a}}{\mathrm{RT}}$$

This leads to the following equations for the FWO, STK, and KAS methods, respectively:

(10) $$\log\beta =\log\left[\frac{AE_{a}}{\mathrm{RG}(\alpha )}\right]-2.315-0.4567\left(\frac{E_{a}}{\mathrm{RT}}\right)$$

(11) $$\ln\left(\frac{\beta }{T^{2}}\right)=\ln\left[\frac{\mathrm{AR}}{E_{a}g(\alpha )}\right]-\frac{E_{a}}{\mathrm{RT}}$$

(12) $$\ln\left(\frac{\beta }{T^{1.92}}\right)=\mathrm{Cs}-1.0008\frac{E_{a}}{\mathrm{RT}}$$

The Friedman method was included alongside the integral methods (FWO, KAS, Starink) because, being a differential method, it involves no mathematical approximation of the temperature integral and is therefore not subject to the systematic approximation error inherent to integral isoconversional methods. This makes it a valuable internal consistency check: close agreement between the Friedman and integral-method E_a_–α trends supports the reliability of the extracted activation energies, while systematic divergence is observed at intermediate conversion for the HCS-rich blends. It can itself be diagnostic of overlapping or multi-step decomposition behavior not captured by the integral methods [23]. We acknowledge, however, that the Friedman method is comparatively more sensitive to experimental noise because it relies on the instantaneous reaction rate (dα/dt), typically obtained by numerical differentiation of the TG data [26]. To mitigate this sensitivity, the raw TG data were smoothed prior to differentiation using a moving-average filter. Furthermore, because each α value was regressed across five heating rates (5–25 °C/min) rather than a single run, the least-squares fitting itself provides some averaging of noise across the dataset.

### 2.6. Thermodynamics Analysis

The thermodynamic characteristics of pyrolytic decomposition, the pre-exponential factor (A), the activation enthalpy (ΔH), the Gibbs free energy of activation (ΔG), and the activation entropy (ΔS) were determined from the activation energies found using the FWO, KAS, and STK isoconversional models. The following standard equations were used to calculate the thermodynamic properties from the activation energies:

*Enthalpy changes* Δ*H* = *E_a_* − *RT_m_* (13)

(14) $$\mathrm{Entropy}\;\;\mathrm{changes}\;\Delta S=\frac{\Delta H-\Delta G}{T_{m}}$$

(15) $$\mathrm{Gibbs}\;\;\mathrm{Free}\;\;\mathrm{Energy}\;\;\Delta G=E_{a}+RT_{m}\ln(\frac{K_{B}T_{m}}{\mathrm{hA}})$$

Here, E_a_ denotes the apparent activation energy (kJ·mol^−1^) at a given conversion (α), as determined by the FWO, KAS, or STK isoconversional method; R represents the universal gas constant; T_m_ is the peak temperature (K) at which the DTG curve reaches its maximum decomposition rate for each heating rate; A is the pre-exponential factor (min^−1^) obtained from the same isoconversional method; KB is the Boltzmann constant; and h is Planck’s constant. The units of ΔH, ΔS, and ΔG are kJ·mol^−1^, J·mol^−1^·K^−1^, and kJ·mol^−1^, respectively.

It should be emphasized that ΔH, ΔG, and ΔS calculated via Eyring/activated-complex theory represent the thermodynamic parameters of the reactant-to-activated-complex (transition-state) step specifically, and not the thermodynamics of the overall reactant-to-final-product transformation. As such, these parameters characterize the energy barrier and the degree of order of the transition state relative to the reactant, rather than the net enthalpy or spontaneity of the complete pyrolysis reaction.

## 3. Results

### 3.1. Thermogravimetric Analysis

**Figure 1:**
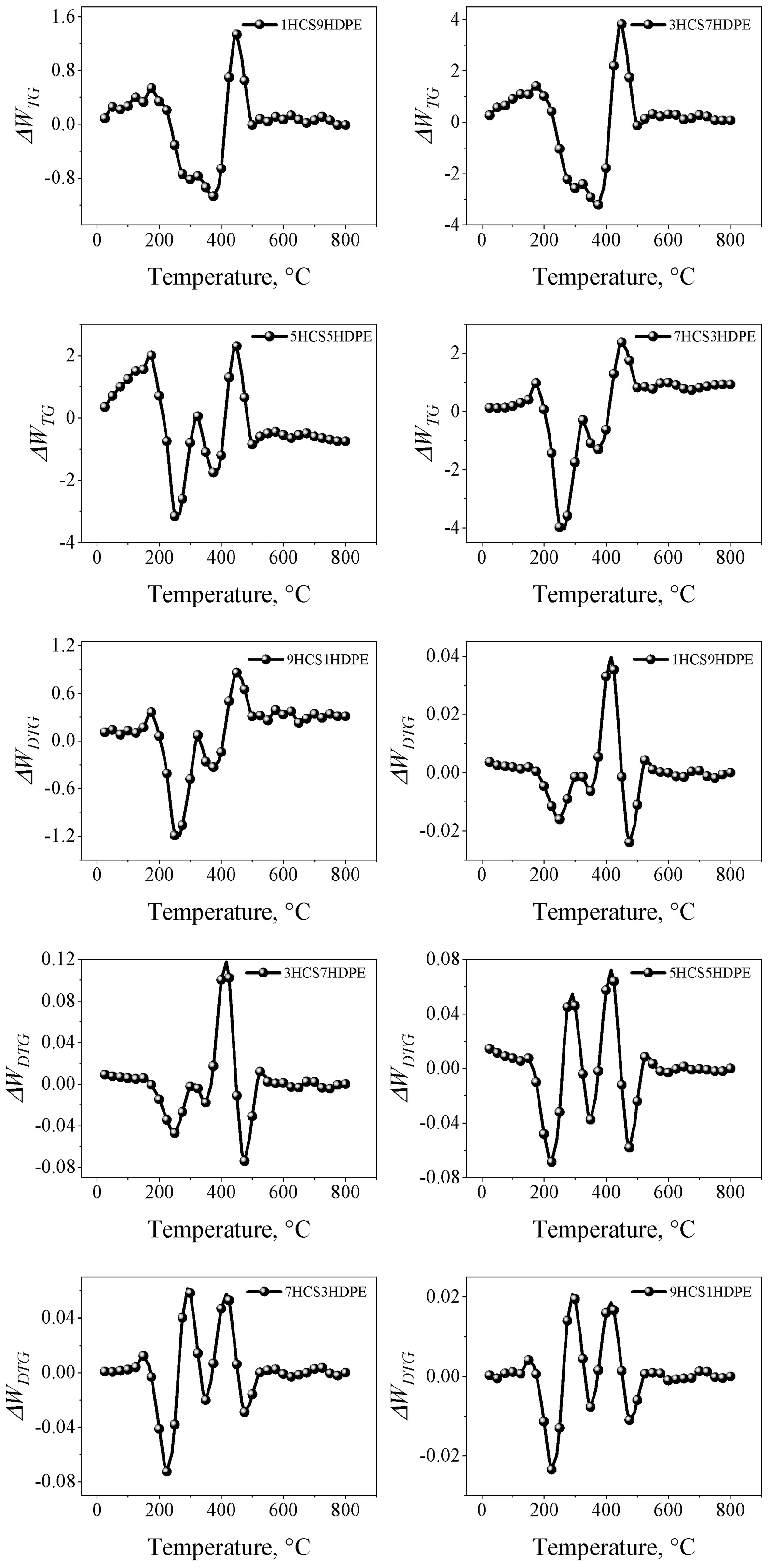
∆W profile of HCS-HDPE blends.

TG and DTG curves obtained for HDPE and hydrochars derived from *Camellia oleifera* shells (HCS) as well as blends of these (1HCS9HDPE, 3HCS7HDPE, 5HCS5HDPE, 7HCS3HDPE, 9HCS1HDPE) all show a similar general shape (Figure 1). This shape consists of (i) a minor mass loss stage below about 150 °C due to residual moisture and light volatiles, (ii) a major stage of volatiles removal, and (iii) a slow tail of mass loss extending to 800 °C related to cracking of secondary chars and progressive carbonization. This staged pattern is consistent with what has been widely reported for residues of lignocellulose and single and blended pyrolysis [10,27]. By itself, HCS, which is hydrochar rather than raw biomass, shows a stage of volatiles removal that is narrower and less intense than would be expected for untreated shells of Camellia oleifer. This is consistent with known effects of hydrothermal carbonization (HTC), which removes a large fraction of hemicellulose and some extractives during the hydrothermal step [28]. Reviews consistently report that biomass treated directly and hydrochar separately react less and yield more char during subsequent thermal treatment because labile hemicellulose and amorphous cellulose fractions have already been hydrolyzed and solubilized into process liquid during HTC [29]. That is why HCS alone produced a flat TG curve with high residue compared to sharp near-complete decomposition reported elsewhere for raw Camellia shell [12,30].

#### 3.1.1. Individual Pyrolysis Behavior of HDPE and HCS

HDPE decomposes into a sharp DTG peak in the ~420 to 500 °C range, as shown in Figure 1. This reflects random cleavage of chains in polyolefins which proceeds through a very narrow temperature window once backbone carbon and carbon cleaves. This single stage and high rate of weight loss is a classic signature reported in many co-pyrolysis studies [10,31,32] and stands sharply in contrast to the broader profile of decomposition of hydrochar from lignocellulose which degrades over a wider temperature range with a much lower maximum mass loss rate [12]. Much larger solid residue (around 15 to 25%) retained by HCS at 800 degrees compared to near-complete volatilization of HDPE is due to higher content of lignin and minerals typical of shell residues favoring aromatization reactions over full volatilization [30,33].

#### 3.1.2. Co-Pyrolysis Blends: Evidence of Synergistic Interaction

Blends containing increasing HDPE fractions show intermediate behavior that goes beyond simple averaging of parent curves, as shown in Figure 1. As HDPE content increases, DTG curves develop sharper high-temperature peaks characteristic of HDPE superimposed on broader envelopes of hydrochar decomposition and final residue decreases systematically with increasing plastic content. This intermediate composition-dependent behavior is a hallmark of synergistic interaction during co-pyrolysis of biomass and HDPE and other polyolefins [10,27,31]. Mechanistically hydrogen transfer is generally attributed between feedstocks: radicals with donating hydrogen and oxygen intermediates are released at low temperature during biomass pyrolysis which stabilize free radicals derived from chain scission of HDPE. On the other hand radicals rich in hydrogen from plastic decomposition promote cracking of tar derived from biomass and hinder repolymerization and char-forming reactions thereby reducing overall solid residue compared to the calculated additive value [10,34,35]. Mechanisms for hydrogen donation have also been proposed specifically for co-pyrolysis of camellia shells and plastic waste. Interaction between these two feedstocks changes both profiles of thermal decomposition and distribution of vapors compared to each component alone. Reviews of co-pyrolysis of biomass and polyolefins also note that synergy depends on composition and generally peaks at intermediate blending ratios (commonly reported in the 20 to 50 weight percent of plastic) before synergy diminishes as plastic becomes so high that molten polymer coats biomass particles and hinders heat transfer which can result in antagonism at very high loading of plastic [35,36].

Reduction observed in residue at the end relative to the sum of TG curves for blends of HCS and HDPE is consistent with this framework and broadly aligns with consensus that plastics rich in hydrogen, such as HDPE, promote deeper volatilization of lignocellulose components, and this results in lower biochar yield but higher yield of volatiles and bio-oil [31,35,37].

To quantify the synergistic interaction, the deviation between the experimental and mass-weighted calculated TG and DTG curves (ΔW*_TG_* and ΔW*_DTG_*, respectively) was evaluated as a function of temperature for all five blends (Figure 1; underlying experimental and calculated curves shown in Figure S1). Rather than a simple, uniform offset, all blends displayed a distinctive, temperature-dependent sign change in ΔWTG: a small positive deviation at low temperature less than 200 °C, indicating marginally less mass loss than predicted, followed by a pronounced negative deviation in the 200–400 °C range (ΔWTG reaching approximately −1 to −4 depending on blend composition, indicating substantially enhanced mass loss relative to the additive prediction), and finally a sharp positive deviation coinciding with the main HDPE decomposition region (400–480 °C; ΔWTG), before the deviation converged toward zero above ~500 °C once devolatilization was essentially complete in both the experimental and calculated curves.

The negative ΔWTG region (200–400 °C) coincides with the principal devolatilization range of HCS, indicating that hydrochar decomposition is accelerated in the presence of HDPE beyond what simple additive behavior would predict, consistent with early-stage hydrogen-radical transfer from softening/pre-decomposing HDPE stabilizing hydrochar-derived oxygenated radicals and promoting their volatilization. Conversely, the positive ΔWTG region (400–480 °C), which coincides with the principal HDPE decomposition stage, indicates that HDPE mass loss is somewhat suppressed relative to the additive prediction at this stage, suggesting that the earlier hydrogen-donation event partially depletes the hydrogen pool otherwise available to sustain HDPE’s own unimpeded random-scission decomposition, or that some radical recombination/stabilization involving hydrochar-derived char fragments moderates the rate of HDPE volatilization once its principal decomposition regime is reached. A qualitatively similar two-stage (enhancement-then-suppression) deviation pattern was observed in the corresponding ΔWDTG curves, confirming that this behavior reflects a genuine, rate-level kinetic interaction rather than an artifact of the cumulative (integral) TG comparison alone.

The magnitude of both the negative and positive deviation regions scaled with HCS content up to intermediate ratios, peaking at 5HCS5HDPE and 7HCS3HDPE (ΔWTG), before diminishing again at 9HCS1HDPE, consistent with the composition-dependent interaction strength already identified in the kinetic (E_a_–α) and thermodynamic (ΔS–α) analyses. Notably, the experimental DTG curves for the HCS-richer blends (7HCS3HDPE, 9HCS1HDPE; Figure S1) display a distinct secondary shoulder/local maximum in the 250–350 °C range that is not reproduced by the mass-weighted calculated DTG curve, which instead follows a comparatively smoother, single-envelope trend. The appearance of this additional experimental feature rather than merely a shifted or scaled version of the calculated curve provides direct qualitative evidence that co-pyrolysis proceeds through a decomposition pathway distinct from the simple superposition of the two individual feedstocks, reinforcing the synergistic interaction mechanism proposed in Section 3.4.

#### 3.1.3. Effect of Heating Rate

**Figure 2:**
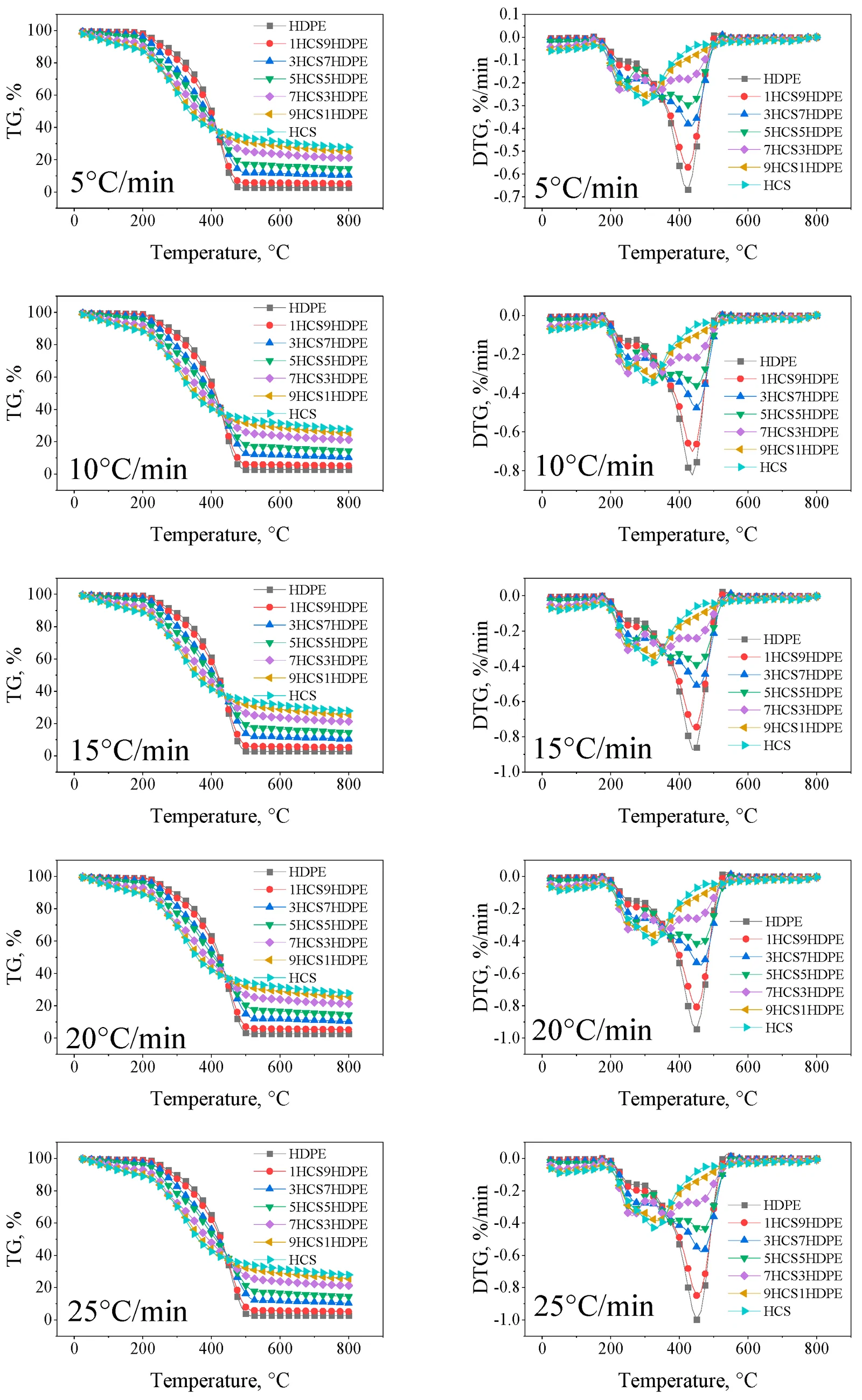
TG and DTG curves of HDPE, HCS, and their blends at different heating rates.

Across blend ratios, heating rate has a clear effect: raising from 5 to 25 °C/min shifts the onset of the main decomposition stage and DTG peak temperatures to higher temperatures and also increases the maximum mass loss rate (making peaks deeper and sharper at higher heating rates), as shown in Figure 2. This is well known: at higher heating rates, there is finite time for heat to penetrate the sample and volatiles to escape particles, so actual sample temperature lags behind programmed furnace temperature. Sharper peaks and rightward shift with heating rate have also been reported separately for lignocellulose biomass [38] and polyolefin plastics [10] and blending of HCS and HDPE follows this same trend: this artifact related to transport of heat dominates over any heating rate-dependent change in chemical interaction between these two. This heating rate sensitivity is why TG-DTG data of this kind generated here serve as a standard basis for free model kinetic analysis (e.g., KAS, FWO and Friedman) that you might want to pursue next to quantify apparent activation energies of blends compared to the weighted average mass of pure HCS and HDPE; this is a common step used to further verify rather than visually infer synergistic effects seen from curves [10,39].

Overall, profiles of TG and DTG show: (i) hydrochar HCS is much more thermally stable and forms char compared to raw lignocellulose biomass, consistent with changes induced by hydrothermal carbonization; (ii) HDPE decomposes through a single sharp stage of high temperature volatilization typical of polyolefins; (iii) blends of co-pyrolysis display DTG profiles that depend on composition and lower solid residues compared to the calculated weighted average, suggesting positive synergistic interaction most plausibly involving transfer of hydrogen from radicals derived from hydrochar and HDPE; and the systematic shifting of peaks to higher temperatures with increasing heating rate reflects limitations of heat and mass transfer rather than change in reaction pathway.

### 3.2. Kinetic Analysis of HCS/HDPE Co-Pyrolysis (Isoconversional Methods)

**Figure 3:**
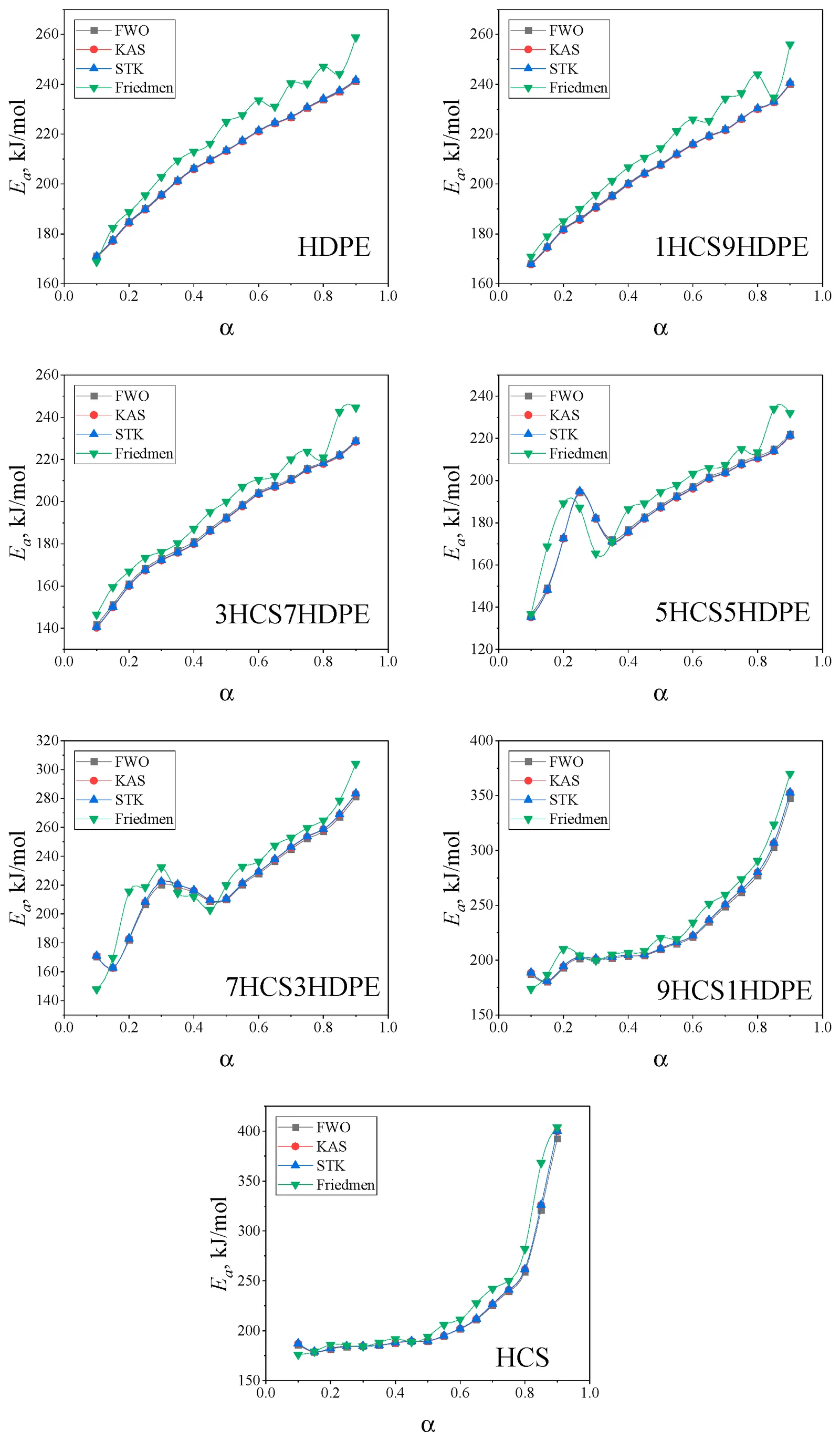
Activation energy (E_a_) vs. conversion (α) by FWO, KAS, STK, and Friedman methods for individuals and blends.

Across samples generally, curves for FWO, KAS and STARINK overlap closely together, but the Friedman curve consistently runs higher and has more scatter, especially at high conversions (Figure 3). This is a well-documented feature of isoconversional analysis: FWO, KAS and Vyazovkin use integral methods sharing the same underlying integral approximation, so they tend to converge on similar trends of E_a_ versus α. Friedman is a differential method that directly uses instantaneous reaction rate and avoids approximation error but is correspondingly more sensitive to experimental noise and genuine kinetics involving multiple steps, which is why it usually reports higher and more fluctuating E_a_ values [40,41]. A consistent divergence pattern, with clustering closely of FWO, KAS and Vyazovkin traces against distinct higher traces of Friedman, has also been consistently reported for both lignocellulose biomass [40] and mixed plastics pyrolysis [42], which supports internal consistency of your dataset.

Pure HDPE E_a_ rises smoothly from roughly 170 kJ/mol at low conversion to roughly 240 kJ/mol at high conversion. This progressive increase follows the accepted mechanism for degradation of polyolefins: as pyrolysis progresses, bonds that are easier to cleave and that are lighter and branched are consumed first. This leaves segments of the backbone that are increasingly stable and scission of which requires higher energy. This manifests as an increasing activation energy E_a_ with α [43,44]. Here we find absolute magnitude to be somewhat lower but broadly comparable to activation energies reported using similar isoconversional methods elsewhere; for neat HDPE pyrolysis, values reported range from 238 to 247 kJ/mol (Friedman/KAS/FWO) [43] and 259 to 270 kJ/mol (FWO/KAS/Starink using two-dimensional nucleation model) [45]. Differences here are not unusual because apparent activation energy is known to be sensitive to sample form (pellets versus powder), particle size and the specific heating rate range used [45].

HCS shows quite a different profile: activation energy E_a_ remains fairly flat around 180 to 200 kJ/mol for low to mid conversions and then rises sharply to around 350 to 400 kJ/mol when α is near 0.8. High conversion upturns recur in isoconversional studies of lignocellulosic and char-like materials and are usually attributed to residual solids becoming richer in recalcitrant aromatic fractions derived from lignin as labile cellulose and hemicellulose are consumed [46,47]. Because HCS is hydrochar rather than raw biomass, it starts out deficient in easily hydrolyzable hemicellulose fractions removed during hydrothermal carbonization. So at high α conversion, material reacting there is disproportionately composed of partially condensed and aromatic structures derived from lignin; these require progressively more energy to break down further because volatilization at this stage competes with and is increasingly dominated by reactions that link and cross-link char rather than releasing volatiles [8,46]. This mechanism is reported widely using the same family of isoconversional methods: rising activation energy at high conversion linked to aromatization of lignin and char [40,47].

Blends, especially 5HCS5HDPE and 7HCS3HDPE, show an unusual dip-and-rise on the E_a_–α curves in the α = 0.2–0.4 region; such dips were not observed (or were much less pronounced) for pure HDPE and pure HCS. Such dips have been interpreted by many authors studying co-pyrolysis of polymers as indicative of a real chemical interaction between polymer types, and not merely additive decomposition behaviors of individual polymers. For example, the E_a_ minimum occurs in the temperature range (T ≈ 550 °C) where the initial softening and cracking of HDPE occur approximately concurrently with the primary devolatilization steps of the hydrochars produced via fast pyrolysis. This has been attributed to hydrogen atoms generated from thermal cracking of HDPE acting as radical stabilizers to slow down biomass-derived radical reactions until they reach temperatures where the “true” high-temperature regimes of HDPE chain scissions dominate, and E_a_ increases rapidly again [48]. Several reviews of biomass-plastic co-pyrolysis synergy have identified the two primary interactions responsible for these non-linear kinetic behaviors as: (1) active hydrogen atom transfer from plastics to biomass-oxygenated radicals and (2) catalysis due to alkali/alkaline earth cations found in biomass ash [8,35]. The increased prominence of this phenomenon at intermediate to high levels of HCS loading (5HCS5HDPE, 7HCS3HDPE), relative to lower HCS loadings, is also consistent with prior studies indicating that synergistic effects can depend on both the ratio of reactants and the specific type(s) of biomass/plastics being reacted [35].

**Figure 4:**
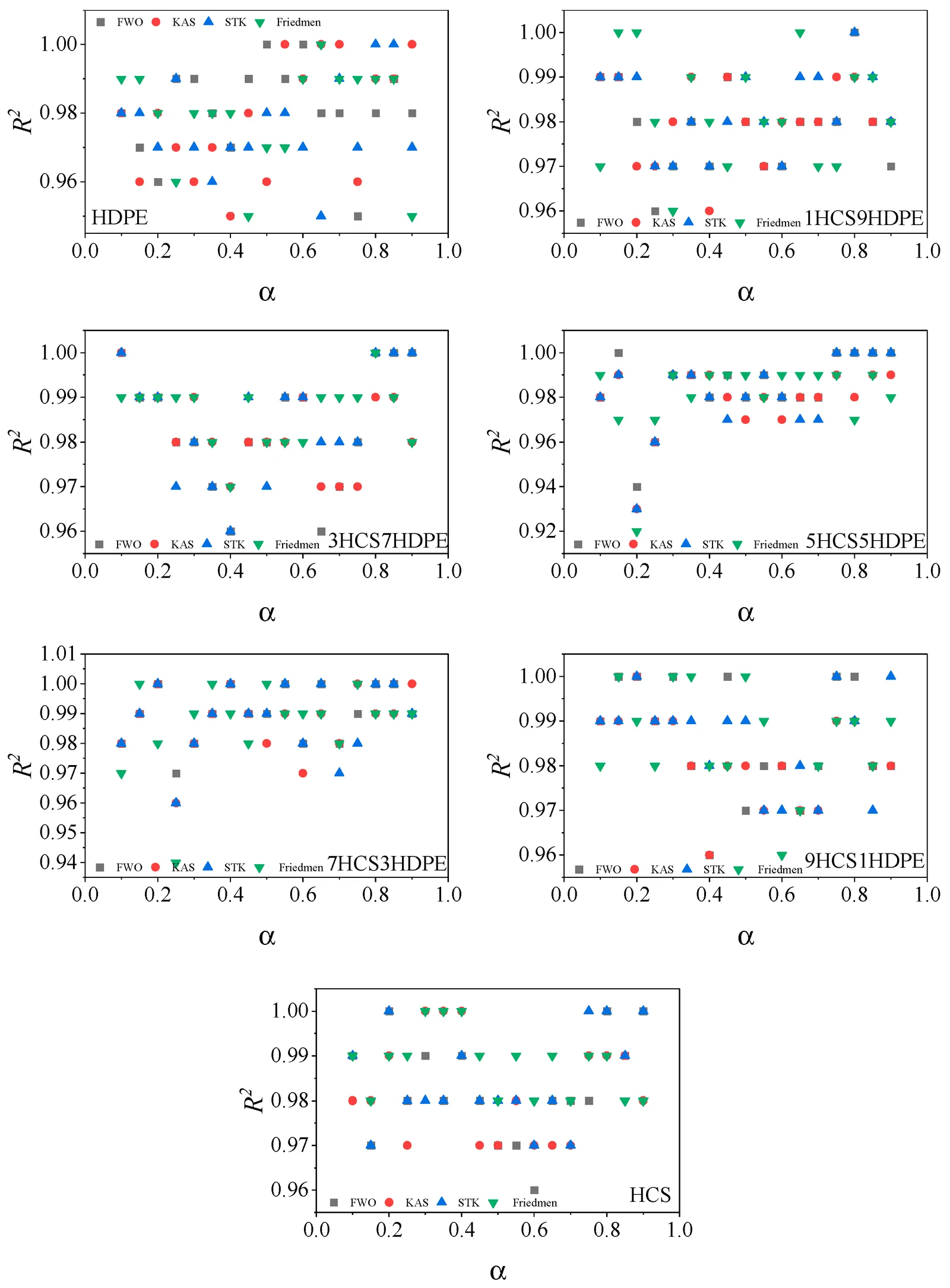
Coefficient of determination (R^2^) vs. conversion (α) for the FWO, KAS, STK, and Friedman methods for individuals and blends.

This large difference in E_a_ values in the high-conversion regime illustrates why collapsing the isoconversional results into a single, conversion-averaged activation energy—a simplification sometimes adopted for convenience elsewhere in the literature—would fail to capture the conversion-dependent complexity of the reaction observed here. Accordingly, no such average was calculated in this study; E_a_ is instead reported explicitly as a function of conversion (α) throughout (Figure 3), consistent with the ICTAC Kinetics Committee recommendations underlying the isoconversional methods used [40,46]. A reasonable degree of agreement among FWO, KAS, and STK methods across the entire series also supports the use of their mean as the “main” E_a_ trend for designing reactors based on kinetic data, and considering Friedman’s higher and noisier trace primarily as a tool to diagnose complex/multi-step kinetics and determine if additional analyses (e.g., differential scanning calorimetry (DSC)) are required. As shown in Figure 4, the coefficient of determination (R^2^) values obtained from the FWO, KAS, and STK methods remained consistently above 0.99 across the entire conversion range (α = 0.1–0.9) for all samples, confirming the high reliability and goodness of fit of the isoconversional linear regressions, whereas the Friedman method showed comparatively lower and more scattered R^2^ values at certain conversion levels, reflecting its greater sensitivity to instantaneous rate fluctuations and multi-step reaction behavior.

### 3.3. Thermodynamic Analysis of HCS/HDPE Co-Pyrolysis

**Figure 5:**
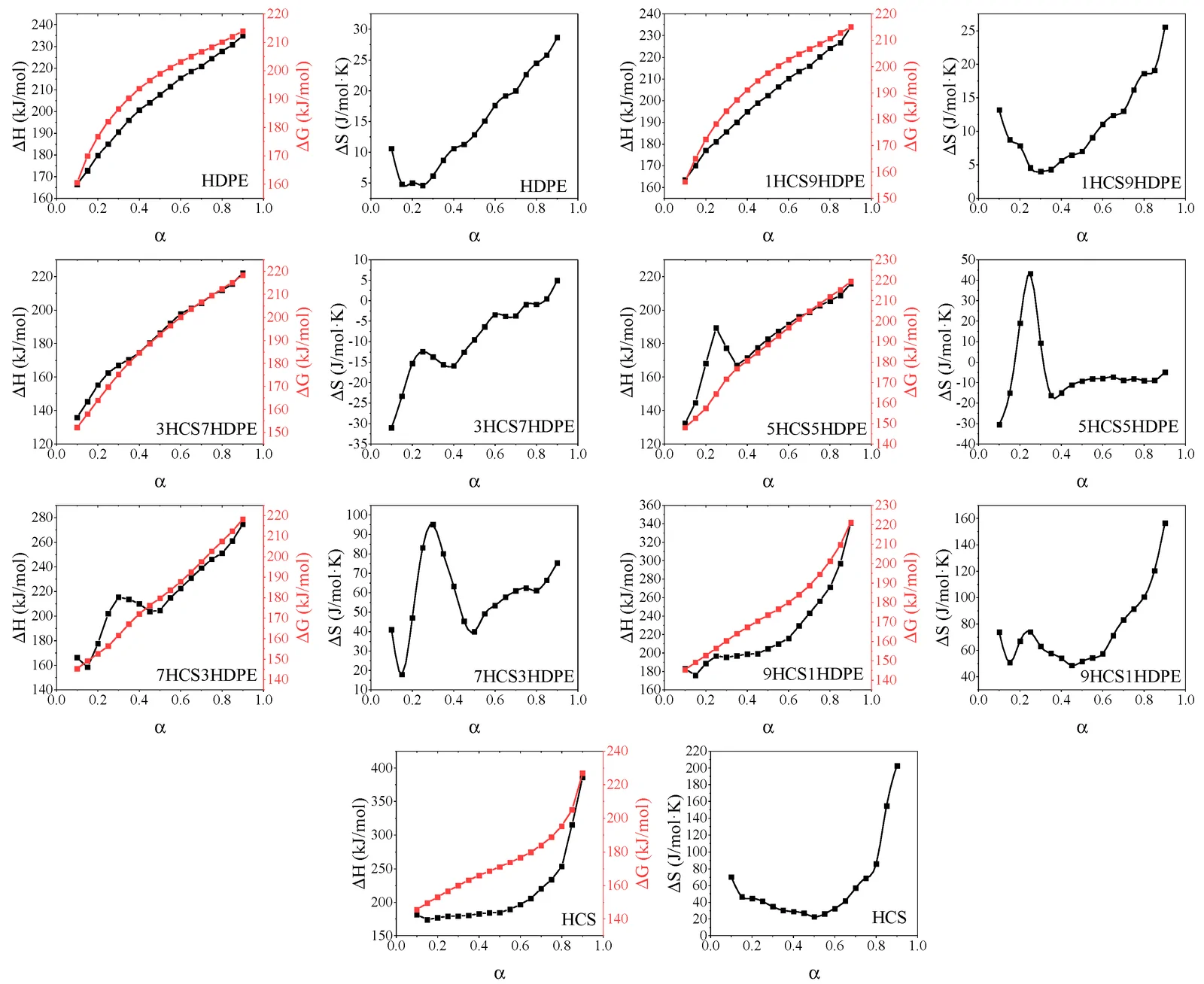
Thermodynamic parameters using FWO, KAS, and STK methods for individual samples and blends.

Enthalpy (ΔH), Gibbs free energy (ΔG), and entropy (ΔS) are determined using isoconversional methods based on activation energy (E_a_) and the pre-exponential factor (A) using Eyring theory: approximately ΔH ≈ E_a_ − RT. ΔG is obtained from the peak characteristic temperature and A while ΔS follows from ΔH and ΔG as shown in Figure 5 [49]. Within this framework, ΔH represents the apparent difference in energy between reactants and the rate-limiting activated complexes governing decomposition at each α. ΔG represents the apparent total energy demand for reactants to reach the rate-limiting activated states (actual energy available or required in the system). It should further be noted that pyrolysis, particularly of a multi-component blend such as HCS/HDPE, proceeds through a series of parallel and sequential elementary reactions (e.g., dehydration, decarboxylation, chain scission, aromatization) rather than a single reaction step. The ΔH, ΔG, and ΔS values reported at each conversion degree α are therefore apparent (effective) activation parameters, reflecting the composite behavior of whichever reactions dominate the overall decomposition rate at that specific α, rather than the thermodynamic properties of one specific, molecularly defined activated complex consistent with the model-free premise of the isoconversional kinetic methods (Section 2.6) that similarly yield an apparent activation energy rather than resolving the parameters of individual elementary steps [50]. Consistent with this distinction, ΔH and ΔG throughout this section are denoted ΔH and ΔG to explicitly signal that they describe the activation step, not the overall reactant-to-product transformation.

Across samples HDPE and HCS and all blends, ΔH is consistently positive throughout the conversion range and closely tracks the trend of E_a_ (deviating only by a small RT term that is typically a few kJ/mol at pyrolysis temperatures), as expected because ΔH = E_a_ − RT [51]. The consistent positivity of ΔH indicates that a substantial apparent energy barrier must be overcome to reach the rate-limiting activated states at every stage of conversion, requiring continuous external heat input to sustain decomposition, a finding at the level of the activation step that is consistent with reports for single biomass pyrolysis [52] and systems co-pyrolyzing biomass and plastics, though it should not be read as characterizing the thermodynamics of the overall reactant-to-product reaction [53,54]. Rise in ΔH with α mirrors the rise in E_a_ discussed before for these samples, and especially for HCS there is a sharp rise late in conversion (up to about 380 kJ/mol near α close to 0.9) that reflects greater energy demand breaking down residual highly aromatic hydrochar skeletons derived from lignin at high conversion.

ΔG remains consistently positive and stable throughout the whole range of conversion across all samples (roughly 160 to 220 kJ per mole). It rises only slightly compared to the steep trends of ΔH/E_a_; this pattern is well reported in thermodynamic studies of pyrolysis of biomass and mixtures of biomass and plastics, where ΔG changes little with reaction stage because it aggregates compensating effects of E_a_ and pre exponential factor A rather than reflecting chemistry sensitively like E_a_ does [52,55]. The consistent positivity of ΔG across all ratios of HCS to HDPE indicates that formation of the rate-limiting activated complexes requires additional free-energy input at every blend composition, i.e., the activation step does not proceed spontaneously without external heat input; a similar interpretation, at the level of the activation step, has been drawn for other mixed biomass–plastic systems (such as date pits with polypropylene and polystyrene), where positive ΔH and ΔG together were used to describe an energetically demanding, externally heat-driven activation process [53]. We note that this interpretation applies specifically to the activation step evaluated via transition-state theory and does not establish the spontaneity or thermodynamic sign of the overall, non-isothermal reactant-to-product pyrolysis reaction, for which the classical spontaneity criterion (ΔG < 0) is strictly defined only under isobaric–isothermal conditions. ΔH and ΔG show a relatively small and consistent gap, typically on the order of just a few tens of kJ/mol, which further indicates that once activation by heat occurs, the reaction proceeds readily toward products rather than reverting: this has also been reported for other feedstocks of lignocellulose where ΔH minus ΔG stays narrow through multistep decomposition [51,56].

ΔS is an especially interesting diagnostic because unlike ΔH and ΔG, it directly reflects how ordered or loose the rate-limiting activated complexes are compared to the reactant: negative ΔS means that the activated complex is more ordered and compact, and this correlates with lower reactivity and a slower step of reaction; positive ΔS means that the activated complex is more disordered and closer to dissociation, and this correlates with higher reactivity [57]. Looking at data through this lens HDPE shows ΔS approaching near zero or slightly positive at α close to 0.2 and then steadily rising to strongly positive values around 25 J/mol·K at high α.

HCS shows negative to near-zero entropy across most of the low and mid conversion range and then rises sharply to strongly positive values around α above 0.8. A transition from negative entropy at low conversion to strongly positive at high conversion has specifically been reported for lignocellulosic residues and interpreted as reflecting sequential breakdown of hemicellulose, cellulose and finally lignin types of structures. Late stages release larger numbers of smaller and more disordered volatile fragments once highly cross-linked aromatic networks finally break down [56].

Blends show pronounced ΔS peaks and troughs (oscillating roughly between −30 and +90 J/mol·K) roughly around the same intermediate conversion range (α around 0.2 to 0.4) where characteristic fluctuations are seen in Eα vs. α curves for these blends as well. Because ΔS is derived from the same A and E_a_ values, this parallel behavior is expected, and it also supports earlier interpretation: transiently more disordered complexes form during this conversion window and disrupt normal decomposition pathways involving HDPE and hydrochar intermediates [8]. Overall, these results are internally consistent with the kinetic picture: positive ΔH and ΔG across compositions indicate an energetically demanding, externally heat-driven activation step for pyrolysis of HDPE, hydrochar, and their blends, describing the transition-state energetics rather than the overall reaction thermodynamics [51,53]; a narrow and stable gap for ΔH and ΔG suggests efficient thermal activation; strongly conversion- and composition-dependent ΔS especially sharp excursions in blends of HCS and HDPE corroborate the inferred pathway of multi steps driven by interactions [8,57].

### 3.4. Proposed Mechanism of HCS/HDPE Co-Pyrolysis

**Figure 6:**
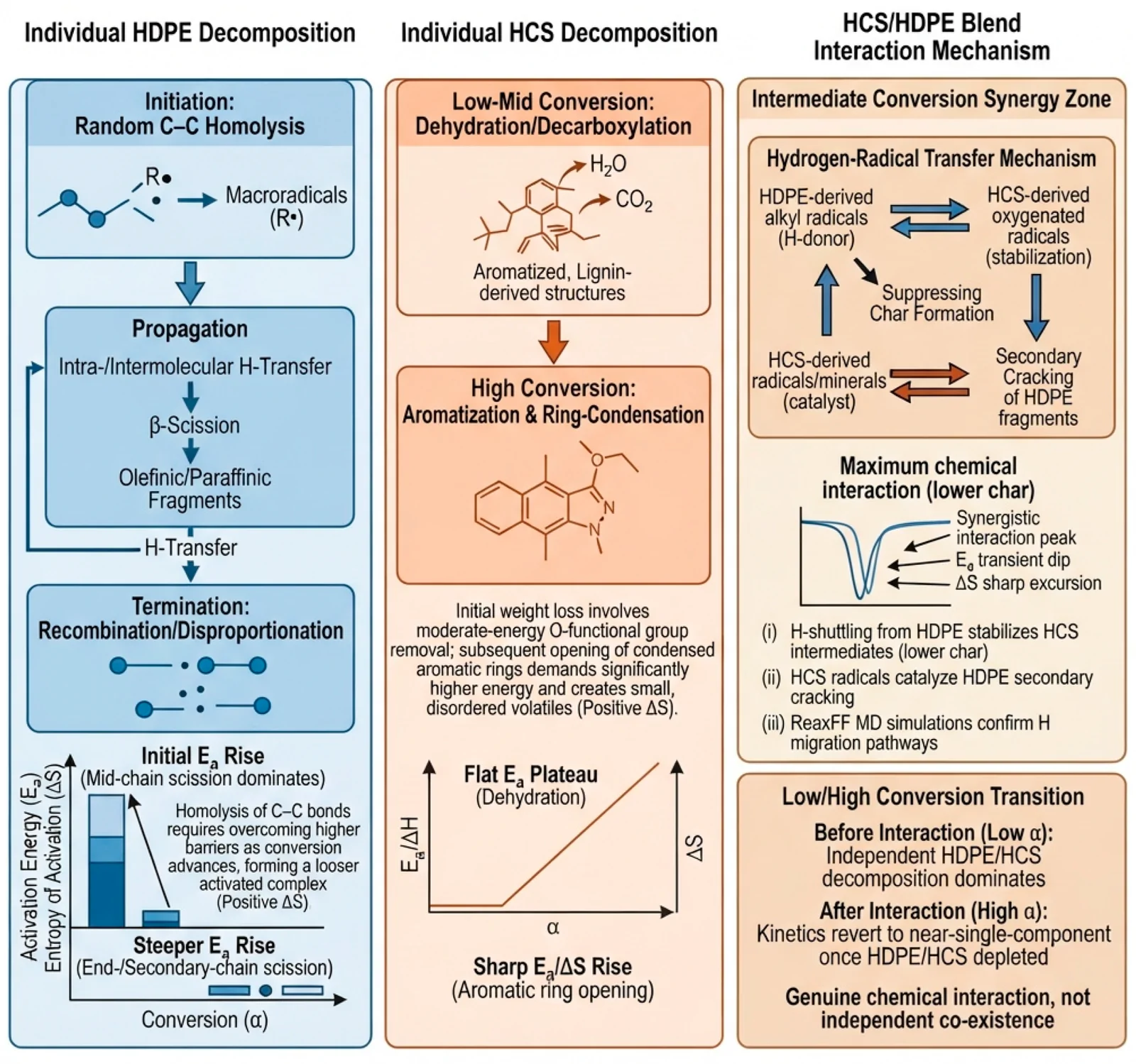
Proposed mechanism of HCS/HDPE Co-pyrolysis.

HDPE degrades through the free-radical chain process: Homolysis at random locations within the polymer backbone produces macroradicals, which then can be stabilized via intra-/inter-molecular hydrogen abstraction, and subsequently undergo beta-scission producing olefinic/paraffinic fragments, ultimately being terminated by combination or disproportionation [58,59]. As the degree of reaction increases, there will be a gradual increase in the proportion of secondary and chain end radicals involved in the degradation, and because these are formed by overcoming an increased energy barrier, they explain the increasing values of E_a_ and the increasing positive value of ∆S seen for HDPE. The formation of the activated complex for the latter stage degradation is less ordered and more dissociative (Figure 6), thus explaining why the entropy of activation for this bond-breaking step would also be positive [58,59].

Since HCS was previously hydrothermally treated, much of its labile hemicellulose had already undergone hydrolysis, dehydration and decarboxylation to produce a hydrocarbon enriched in partially aromatized structures derived from lignin [12,13]. Therefore, pyrolysis of this structure occurs through two separate mechanistic steps: low–mid conversion dehydration–decarboxylation of the remaining oxygen-containing groups; high conversion aromatization and ring condensation of the increasingly refractory carbon skeleton due to decarboxylation of volatile fragments. Since the cleavage of bonded aromatic carbon atoms is inherently more difficult than the cleavage of aliphatic or glycosidic linkages, this accounts for the relatively constant Energy of Activation/∆ Entropy Plateau observed at low–mid alpha values and the sharp upward spike in Energy of Activation/∆ Entropy observed at late conversions (simultaneous with a peak in ∆ S indicating the release of volatile fragments into the vapor phase as the aromatic structure eventually begins to open) [13,60].

The fact that both DTG profiles exhibit non-additive behavior and that there are significant fluctuations in E_a_ and ∆S at intermediate conversion in both 5HCS5HDPE and 7HCS3HDPE blend samples indicates that there exists a true chemical interaction occurring between the two materials rather than simply their independent simultaneous decomposition. Based on this evidence, together with mechanisms reported in the biomass–polyolefin co-pyrolysis literature, we propose that the general mechanism for the pyrolytic co-decomposition of biomass-plastics is hydrogen radical transfer. As HDPE softens due to its thermal degradation it generates hydrogen-rich alkyl radicals which may diffuse to and stabilize unstable oxygen-containing radicals generated during hydrochar devolatilization, providing an in situ source of hydrogen to prevent repolymerization/char-forming processes; conversely, oxygenated and mineral (alkali/alkaline earth) catalyzed radicals generated from the hydrochar facilitate secondary cracking reactions on HDPE fragments when both decomposition windows overlap [8]. This bidirectional interaction has been shown to occur by ReaxFF molecular dynamics simulation studies of biomass-plastic systems, demonstrating how plastic-derived radicals enhance hydrogen migration to biomass intermediates while decreasing char-forming condensation [61]. This is consistent with: (a) the lower amount of residual char produced in the blends compared to what would be predicted based upon mass-weighted averages of each component; (b) the transient E_a_ dip and large positive ∆S spike observed at intermediate conversion levels indicative of the point of highest interaction; and (c) the return to nearly single-component kinetic behaviors after either one of the individual material decomposition processes has proceeded sufficiently far [34]. It should be emphasized that this hydrogen-radical transfer mechanism is inferred from the kinetic (E_a_–α) and thermodynamic (ΔS–α) signatures observed in this study, interpreted in the context of mechanistic evidence reported for analogous biomass–polyolefin co-pyrolysis systems [61]. It has not been directly confirmed by spectroscopic, chromatographic, or computational evidence within the present work, and should therefore be regarded as a plausible, the literature-supported hypothesis rather than a proven reaction pathway. Direct verification, for example, via in situ TG-FTIR or TG-MS monitoring of evolved gas species, Py-GC/MS analysis of volatile product distributions, or ReaxFF molecular dynamics simulation of radical interactions specific to the HCS/HDPE system, is recommended as an important direction for future work.

In addition to hydrogen-radical transfer, two further factors intrinsic to HCS composition and structure may contribute to the observed synergy. The alkali and alkaline-earth metals (AAEMs, e.g., K, Na, Ca, Mg) retained in the hydrochar’s ash fraction, briefly noted above as promoters of secondary cracking, are well documented to exert a broader catalytic influence on biomass pyrolysis and biomass–plastic co-pyrolysis, generally lowering primary decomposition temperatures and promoting cracking of volatile intermediates, although their net effect on synergy has been reported to be either promotive or inhibitory depending on AAEM type, concentration, and the specific biomass–plastic system. The HCS ash content in this study was 5.27% (as shown in Table 1); a detailed elemental/mineral speciation (e.g., XRF or ICP-OES analysis of AAEM content) was not performed, and this ash-associated catalytic pathway therefore cannot be precisely resolved from the current dataset. Second, pore structure and the resulting solid–solid/solid–vapor contact behavior may also influence the interaction. Consequently, physical infiltration of molten HDPE into the internal pore network of HCS particles is likely limited, and the interaction between the two phases during co-pyrolysis is more plausibly governed by external particle-surface contact and gas-phase mixing of evolved volatiles within the shared reaction zone, rather than by deep pore-level plastic–char contact. This distinction is consistent with the composition-dependent, rather than uniformly enhanced, nature of the synergy observed across the blending series, since gas-phase and surface-contact-mediated interactions would be expected to scale with the relative proportion and spatial distribution of each component rather than with hydrochar internal pore volume. The strength of the interaction between HCS and HDPE observed in this study is also expected to depend on the degree of dispersion and quality of mixing achieved between the two solid components prior to pyrolysis. In this study, HDPE and HCS were dry-mixed using a vortex mixer to promote homogeneous particle distribution prior to pyrolysis; however, because the two materials remained as physically distinct solid particles rather than being combined into a homogeneous solution or co-precipitated composite, contact between the two phases during heating is still governed primarily by particle-scale proximity and, once HDPE softens, by the extent to which the molten polymer wets or coats adjacent HCS particles. Even with effective initial mixing, factors such as particle size mismatch or localized agglomeration could still influence the effective contact area available for radical exchange, and by extension the strength of the observed synergistic effect. This contact-dependence may help explain why the interaction signature was most pronounced at intermediate, balanced blending ratios (5HCS5HDPE, 7HCS3HDPE) rather than at the extremes, since more balanced mixtures likely provide more heterogeneous particle-particle contact points per unit mass. We acknowledge that this study did not directly quantify dispersion uniformity (e.g., via SEM imaging of the blend or post-pyrolysis residues), and recommend this as a valuable direction for future work, alongside the textural characterization noted above.

## 4. Conclusions

This work assessed the co-pyrolysis behavior, kinetics, thermodynamics, and reaction mechanism of *Camellia oleifera* shell hydrochar (HCS) and HDPE across the full blend range. Proximate and ultimate analyses showed that hydrothermal carbonization upgraded raw shell into a higher-carbon, higher-heating-value product (HHV rising from 18.07 to 20.80 MJ/kg), consistent with dehydration, decarboxylation, and partial aromatization during treatment. TG-DTG results showed HDPE decomposing through a single sharp high-temperature stage typical of random-scission polyolefin pyrolysis, while HCS decomposed over a broader range and left a much larger char residue, reflecting its lignin-enriched, partially carbonized structure. Co-pyrolyzing the two produced non-additive TG-DTG profiles and lower-than-expected char yields, pointing to a genuine synergistic interaction rather than independent decomposition.

Isoconversional kinetics (FWO, KAS, STK, Friedman) showed activation energy rising steadily with conversion for HDPE, a plateau-then-sharp-rise pattern for HCS, and marked non-monotonic fluctuations in the intermediate blends especially 5HCS5HDPE and 7HCS3HDPE indicating that the interaction strength depends on blend composition and peaks at balanced ratios. Thermodynamic analysis confirmed uniformly positive ΔH and ΔG throughout, consistent with an endothermic, non-spontaneous, heat-driven process, while ΔS mirrored the same conversion-dependent fluctuations, reinforcing the presence of a transient, more disordered activated complex during the interaction. A hydrogen-radical transfer mechanism in which HDPE-derived radicals stabilize oxygenated hydrochar radicals, and vice versa, is proposed as the main driver of this synergy, consistent with the reduced char yield and non-additive kinetic/thermodynamic signatures observed.

Overall, HCS/HDPE co-pyrolysis appears thermally and kinetically favorable for combined agricultural and plastic-waste valorization, with the 5:5 and 7:3 HCS:HDPE ratios showing the clearest synergy and thus the best targets for further optimization. Future work should examine actual product yields and compositions (bio-oil, gas, char) at these favorable ratios, and confirm the proposed hydrogen-transfer mechanism using in situ spectroscopic or computational (e.g., ReaxFF molecular dynamics) approaches.

## Figures and Tables

**Table 1:** Proximate analysis, ultimate analysis, and calculated HHV/LHV.

	Proximate Analysis/%				Ultimate Analysis/%					Calculated Heating Value	
Sample	M	Ash	Volatile	FC *	C	H	O*	N	S	HHV (MJ/kg)	LHV (MJ/kg)
*C. oleifera* shell (CS)	0.53	4.11	70.33	25.03	46.43	5.82	47.42	0.32	0.01	18.07	16.83
HCS		5.27	73.86	20.87	49.45	6.92	43.54	0.09	0.00	20.80	19.33
HDPE	1.05	0.04	98.89	0.02	85.28	14.11	0.00	0.00	0.00	46.40	43.41

* Fixed carbon = 100 − Moisture − Ash − Volatile; O* = 100 − C − H − N − S.

## Data Availability

The original contributions presented in this study are included in the article/Supplementary Material. Further inquiries can be directed to the corresponding author.

## Supplementary Materials

The following supporting information can be downloaded from https://www.mdpi.com/article/10.3390/polym18192423/s1. Figure S1: Experimental and theoretical profile for individuals and their blending samples.
