# Supplementary material for: Decoding the Hydrogen-Transfer Synergy in Camellia oleifera Shell Hydrochar/HDPE Co-Pyrolysis: A Kinetic and Thermodynamic Perspective
Source: Polymers (Basel). 2026 Oct 4;18(19):2423. doi: 10.3390/polym18192423 (PMC13666892; doi:10.3390/polym18192423)
Supplement: Supplementary file 1 [file polymers-18-02423-s001.zip › polymers-4590184-supplementary.pdf]

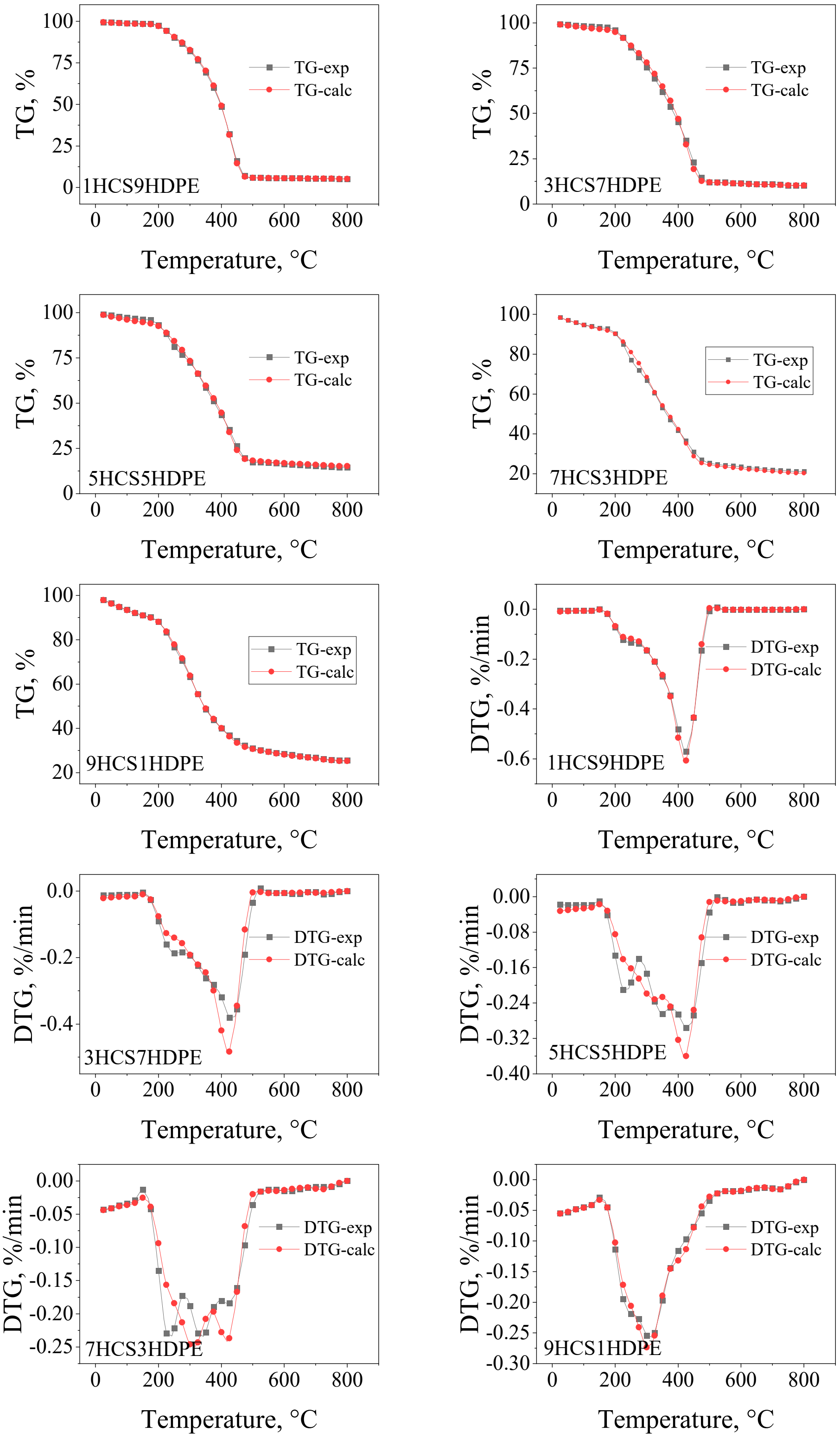

Figure S1. Experimental and theoretical profile for individuals and their blending samples.
